# Co-Circulation of 2 Oropouche Virus Lineages during Outbreak, Amazon Region of Peru, 2023–2024

**DOI:** 10.3201/eid3104.241748

**Published:** 2025-04

**Authors:** Cristhopher D. Cruz, Victoria Espejo, Angelica Espinoza, Roger M. Castillo-Oré, Gilda Troncos, Julia S. Ampuero, Maria E. Silva

**Affiliations:** US Naval Medical Research Unit South, Lima, Peru

**Keywords:** Orthobunyavirus, Oropouche virus, viruses, lineages, vector-borne infections, fever, next-generation sequencing, Peru

## Abstract

We describe introduction of the 2022–2023 Oropouche virus lineage from Brazil, which has caused large-scale outbreaks throughout Brazil, into the Amazon Region of Peru. This lineage is co-circulating with another lineage that was circulating previously. Our findings highlight the need for continued surveillance to monitor Oropouche virus in Peru.

Oropouche fever is caused by Oropouche virus (OROV), a reemerging arthropodborne pathogen endemic in the Amazon Basin. Since its first isolation in Trinidad and Tobago in 1955 (*1*), the virus has caused numerous outbreaks in Central and South America, including in Panama, Trinidad and Tobago, Colombia, Ecuador, Brazil, and Peru (*2*). In recent years, OROV outbreaks have been reported mainly in South America. Colombia reported 2 independent OROV introduction events near the borders of Ecuador and Peru borders during 2017–2020 (*3*,*4*). French Guiana reported an outbreak in 2020 (*5*). Brazil has experienced large-scale outbreaks since 2022 in 6 states of the Amazon Region (Roraima, Amazonas, Acre, Para, Amapa, and Rondonia) (*6*). In Peru, during January 1–July 20, 2024, outbreaks were reported in 5 departments: Loreto, Ucayali, and Madre de Dios, bordered by Brazil to the east; Huanuco in central Peru; and Tumbes, a coastal department in northwestern Peru bordering Ecuador. Peru registered 290 confirmed cases, the highest number of cases reported in Peru’s history (*6*). We describe the identification of the 2022–2023 Oropouche virus lineage from Brazil that was introduced into Peru in 2023. 

The study protocol was approved by the US Naval Medical Research Unit South Institutional Review Board (protocol no. NMRCD.2010.0010) in compliance with all applicable federal regulations governing the protection of human subjects. As part of clinic-based surveillance for acute febrile illness in Peru, we tested blood and serum samples from enrolled participants by quantitative real-time reverse transcription PCR (qRT-PCR) for dengue virus, pan-alphavirus, pan-flavivirus, group C viruses, OROV, and Guaroa virus. Seven samples from Loreto (n = 4) and Madre de Dios (n = 3) tested positive (cycle threshold ≤30) on an in-house developed OROV-specific qRT-PCR. We then sequenced the samples for molecular characterization.

We performed a new RNA extraction from the original positive serum samples by using the QIAamp Viral RNA Mini Kit (QIAGEN, https://www.qiagen.com), according to the manufacturer’s instructions. We amplified the viral genome by using a modified protocol of sequence-independent single-primer amplification (*7*). We prepared libraries by using the Nextera XT DNA Library Preparation Kit and sequenced them by using MiSeq Reagent Kit version 3 (600-cycle) on the MiSeq platform (all Illumina, https://www.illumina.com), following the manufacturer’s instructions. We quality-trimmed raw reads, removed human host sequences, and then de novo assembled the remaining reads. We identified the resulting contigs from samples as OROV by using a BLASTn search (https://blast.ncbi.nlm.nih.gov). We did not detect any OROV contig from the negative control included as a process control. We deposited all obtained consensus sequences into GenBank (accession nos. PP966964–87).

To determine the relationship between the sequenced OROV strains from the Amazon Region of Peru, we performed maximum-likelihood phylogenetic analysis of the small (Figure), medium, and large segments of the viral genome (Appendix Figures 1, 2). We aligned the 3 segments with representative available OROV sequences from GenBank by using MAFFT version 7.56 (*8*). We generated phylogenetic trees by using a general time reversible with gamma distribution model and assessed them with 1,000 bootstrap replicates using PhyML version 3.3.2 (*9*). Phylogenetic trees of the 3 segments showed that the strains from Loreto grouped with 2017–2020 strains from Colombia and a 2016 strain from Ecuador, whereas the strain from Madre de Dios was closely related to the strains from Brazil that caused a recent large-scale outbreak in 2023.

**Figure:**
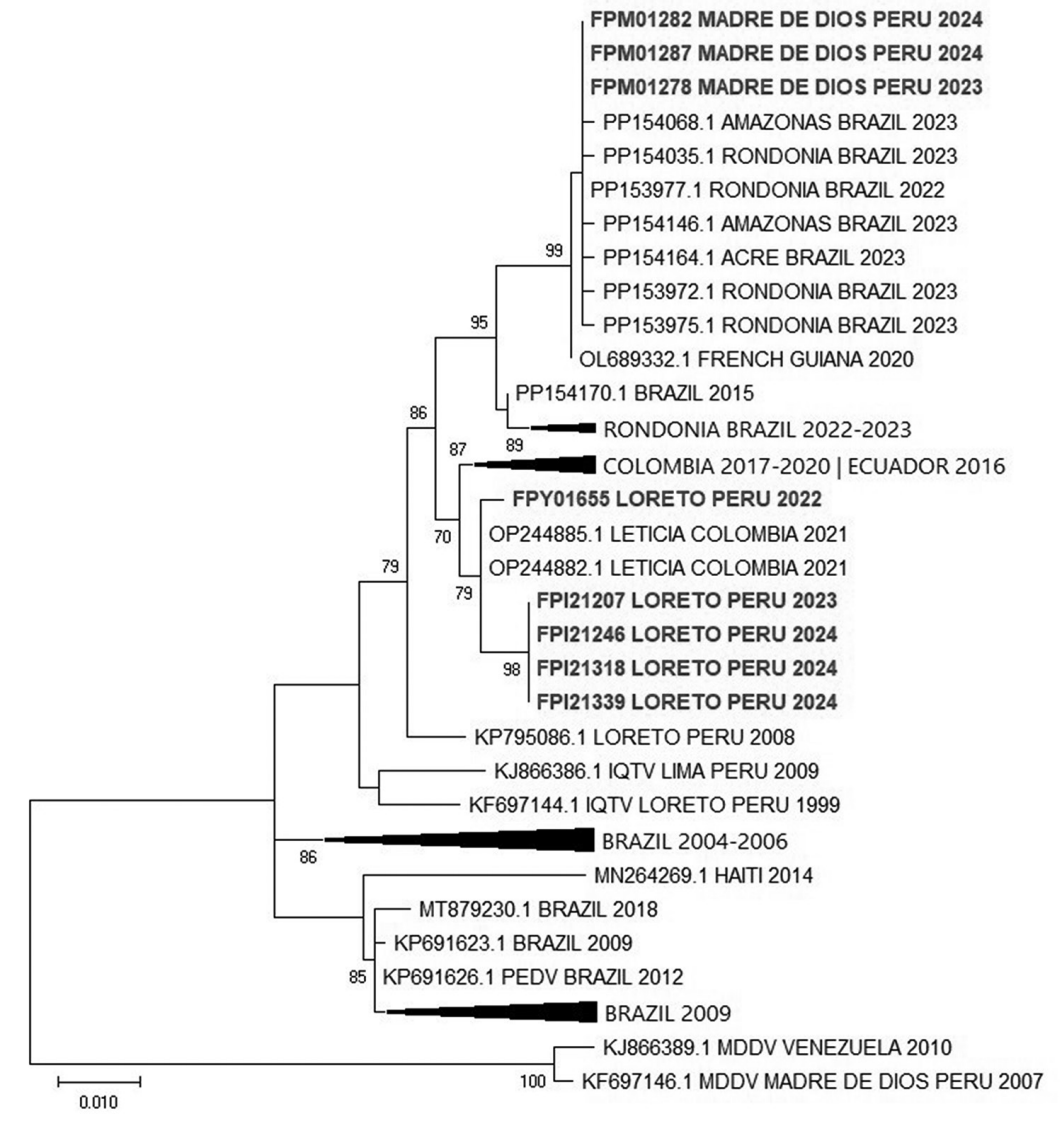
Maximum-likelihood phylogenic tree constructed on the basis of 52 nucleotide sequences of Oropouche virus small segment in study of co-circulation of 2 Oropouche virus lineages during outbreak, Amazon Region of Peru, 2023–2024. Bold type indicates strains from Peru. Only bootstrap values >70% are shown at key nodes. GenBank accession numbers are shown. Scale bar indicates nucleotide substitutions per site.

Our findings indicate that 2 OROV lineages are circulating in the Amazon Region of Peru. One lineage probably was recently introduced through large-scale outbreaks in Brazil, possibly through Madre de Dios, whereas the other is related to strains from Peru, Colombia, and Ecuador that circulated in previous years. Unfortunately, few samples from past outbreaks are available to trace the distribution route of both strains into Peru. Because of the similarity of clinical symptoms between Oropouche fever and other common tropical infectious diseases in the Amazon Region, such as dengue, the use of OROV qRT-PCR in dengue virus–negative samples and high-throughput sequencing can help detect and determine the circulation of OROV in Peru. This approach also will enable prompt identification of the emergence of new or recombined strains that could threaten the civilian and military population living or deployed in affected areas of Peru.

Appendix Additional information about co-circulation of 2 Oropouche virus lineages during outbreak, Amazon Region of Peru, 2023–2024.
