# Supplementary material for: Co-Circulation of 2 Oropouche Virus Lineages during Outbreak, Amazon Region of Peru, 2023–2024
Source: Emerg Infect Dis. 2025 Apr;31(4):879–81. doi: 10.3201/eid3104.241748 (PMC11950249; doi:10.3201/eid3104.241748)
Supplement: Appendix — Additional information about co-circulation of 2 Oropouche virus lineages during outbreak, Amazon Region of Peru, 2023–2024. [file 24-1748-Techapp-s1.pdf]

Article DOI: https://doi.org/10.3201/eid3104.241748

*EID cannot ensure accessibility for supplementary materials supplied by authors. Readers who have difficulty accessing supplementary content should contact the authors for assistance.*

# Co-circulation of Two Oropouche Virus Lineages during Outbreak, Amazon Region of Peru, 2023–2024

## Appendix

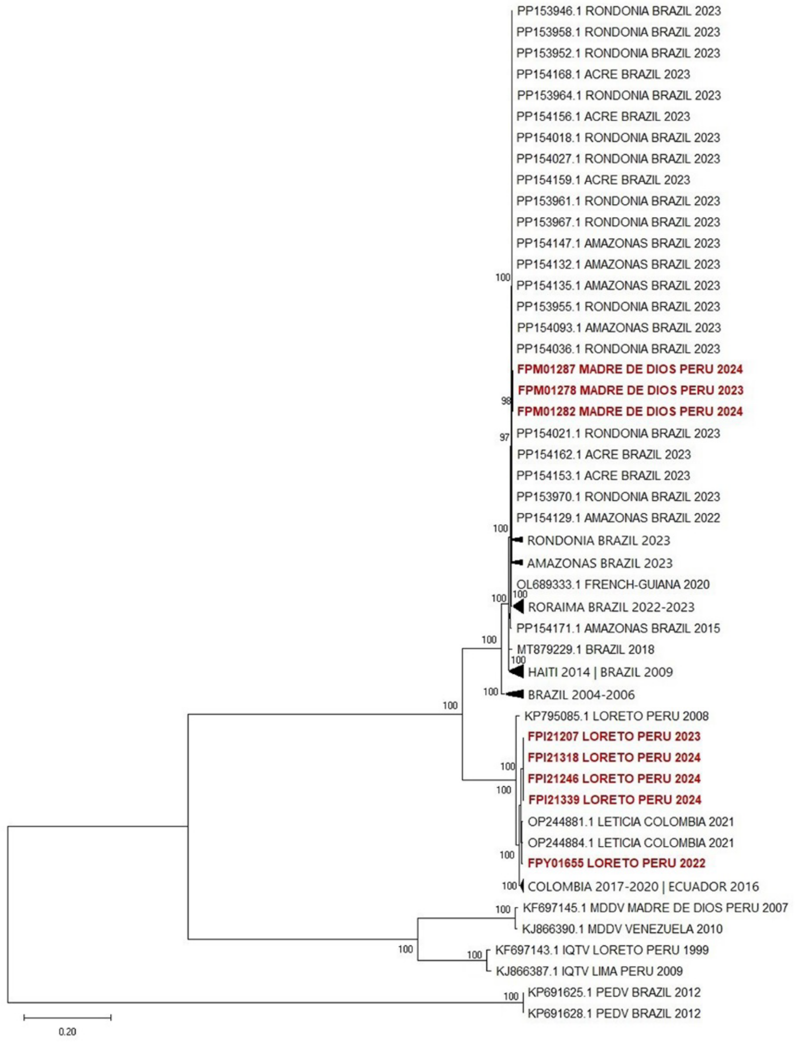


**Appendix Figure 1.** Maximum-likelihood phylogeny based on 78 nt sequences of Oropouche virus M segment. Peruvian strains are highlighted in red bold. Only bootstrap values >70% are shown at key nodes. Scale bar indicates nucleotide substitutions per site.

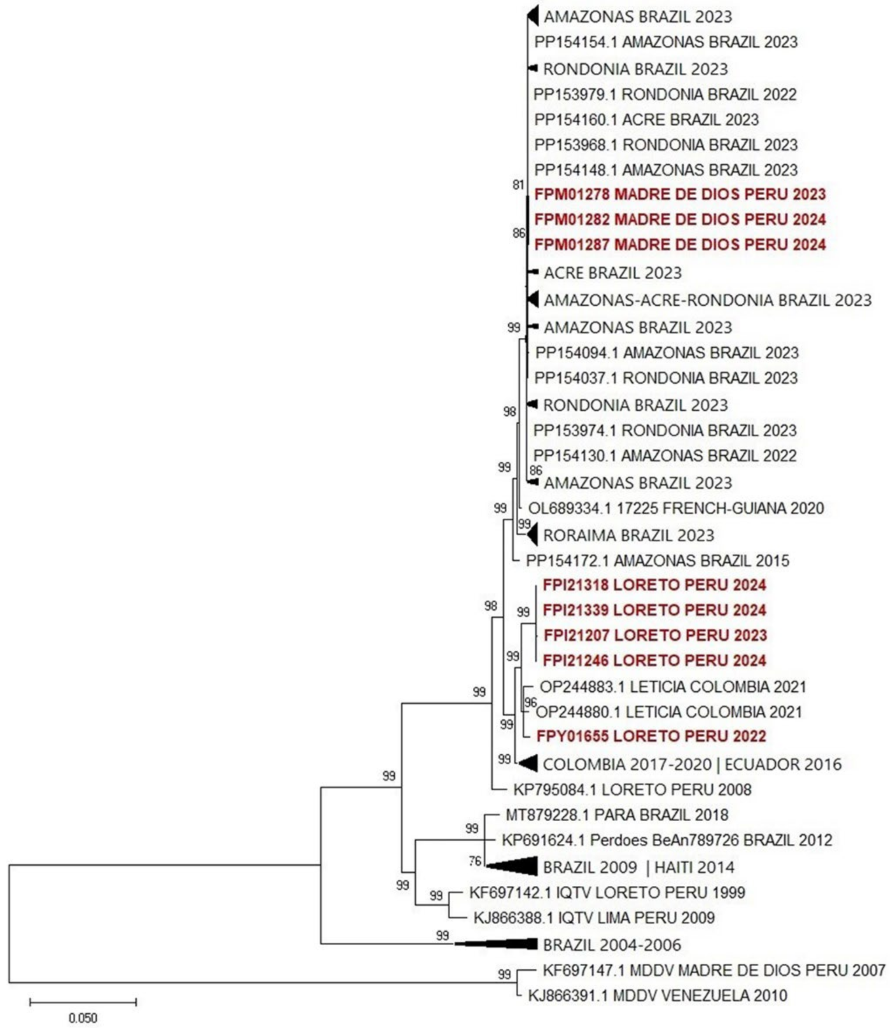


**Appendix Figure 2.** Maximum-likelihood phylogeny based on 93 nt sequences of Oropouche virus L segment. Peruvian strains are highlighted in red bold. Only bootstrap values >70% are shown at key nodes. Scale bar indicates nucleotide substitutions per site.
